# Short-Term Efficacy and Safety of Suvorexant and Lemborexant: A Retrospective Study

**DOI:** 10.7759/cureus.71049

**Published:** 2024-10-08

**Authors:** Koki Mori, Michio Kimura, Eiseki Usami

**Affiliations:** 1 Pharmacy, Ogaki Municipal Hospital, Ogaki, JPN

**Keywords:** falls, lemborexant, orexin, sleep duration, suvorexant

## Abstract

Purpose: Given the risks of long-term benzodiazepine use, safer alternatives like orexin receptor antagonists (ORAs) are needed for insomnia treatment. This study aims to compare suvorexant and lemborexant, focusing on early-stage sleep duration as an efficacy measure and fall incidence as a safety measure.

Methods: We included patients admitted to our hospital between April 1, 2022 and December 31, 2022, who were newly prescribed suvorexant or lemborexant, excluding patients taking other concomitant sleep medications or antipsychotics. Primary and secondary endpoints were sleep duration during the first three days after taking the medications and the incidence of falls, respectively.

Results: We analyzed data from 48 and 57 patients taking suvorexant and lemborexant, respectively. When compared with that in the pre-treatment period, sleep duration was significantly longer on days 2 and 3 in the suvorexant group, and on all three days in the lemborexant group. On day 1 of drug administration, the lemborexant group had a significantly longer sleep duration than the suvorexant group (5.10 ± 1.84 vs. 5.93 ± 1.90 h, respectively; P = 0.017). Zero (0.0%) and three (5.3%) falls occurred in the suvorexant and lemborexant groups, respectively (P = 0.248).

Conclusions:Lemborexant exerted a potent inhibitory effect on orexin 2 receptors, which could explain the longer sleep duration experienced by patients taking this drug on the first day of treatment.

## Introduction

Non-pharmacological therapies [1], such as cognitive behavioral psychotherapy, are recommended as the first-line treatment for insomnia in older patients because of their proven effectiveness. However, patients who do not respond to cognitive behavioral therapy often require pharmacotherapy [2], for which benzodiazepines (BZDs) are among the most commonly prescribed agents. Despite their superior safety to barbiturates, the long-term use of BZDs is associated with an increased risk of cognitive decline [3] due to gamma-aminobutyric acid (GABA) receptor exhaustion, as well as the risk of falls due to muscle relaxation [4]. Additionally, the use of BZDs has been linked to delirium [5] and physical disability [6]; thus, their use is no longer recommended in older individuals [7]. In recent years, medications that do not act on GABA receptors, such as melatonin receptor agonists and orexin receptor antagonists (ORAs), have received considerable attention. ORAs were found to be effective in preventing delirium [8] and are associated with a low incidence of falls [9], making them safer options for treating insomnia.

Although ORAs are safe, the desired response may not be achieved after the first dose [10]. There is also generally a delayed response when cognitive behavioral therapy is used to treat insomnia; therefore, sleep medications with faster onset of action are preferred [11]. Thus, effect immediacy is an important parameter when assessing the efficacy of sleep medication.

Suvorexant [12] and lemborexant [13] are effective ORAs for treating insomnia covered by insurance in Japan. The inhibition of orexin receptors, known to affect arousal, produces sleep effects. Both types of orexin receptors, orexin type 1 receptor (OX1R) and orexin type 2 receptor (OX2R) affect sleep [14]. Suvorexant and lemborexant possess distinct pharmacokinetic parameters as well as differing selectivities for OX1R and OX2R [15]. The time to maximum concentration (Tmax) of suvorexant is 2 h, with a half-life (T1/2) of 12 h [16], whereas lemborexant has a Tmax and T1/2 of 1.5 and 18 h, respectively [17].

The pharmacological and pharmacokinetic differences between suvorexant and lemborexant likely lead to varying efficacies and side effects. However, data comparing the effectiveness and safety of these drugs is scarce. In the current study, we aimed to investigate the efficacy of suvorexant and lemborexant using sleep duration in the early stages of treatment as an efficacy index and the incidence of falls as a safety index.

## Materials and methods

Patients

**Figure 1:**
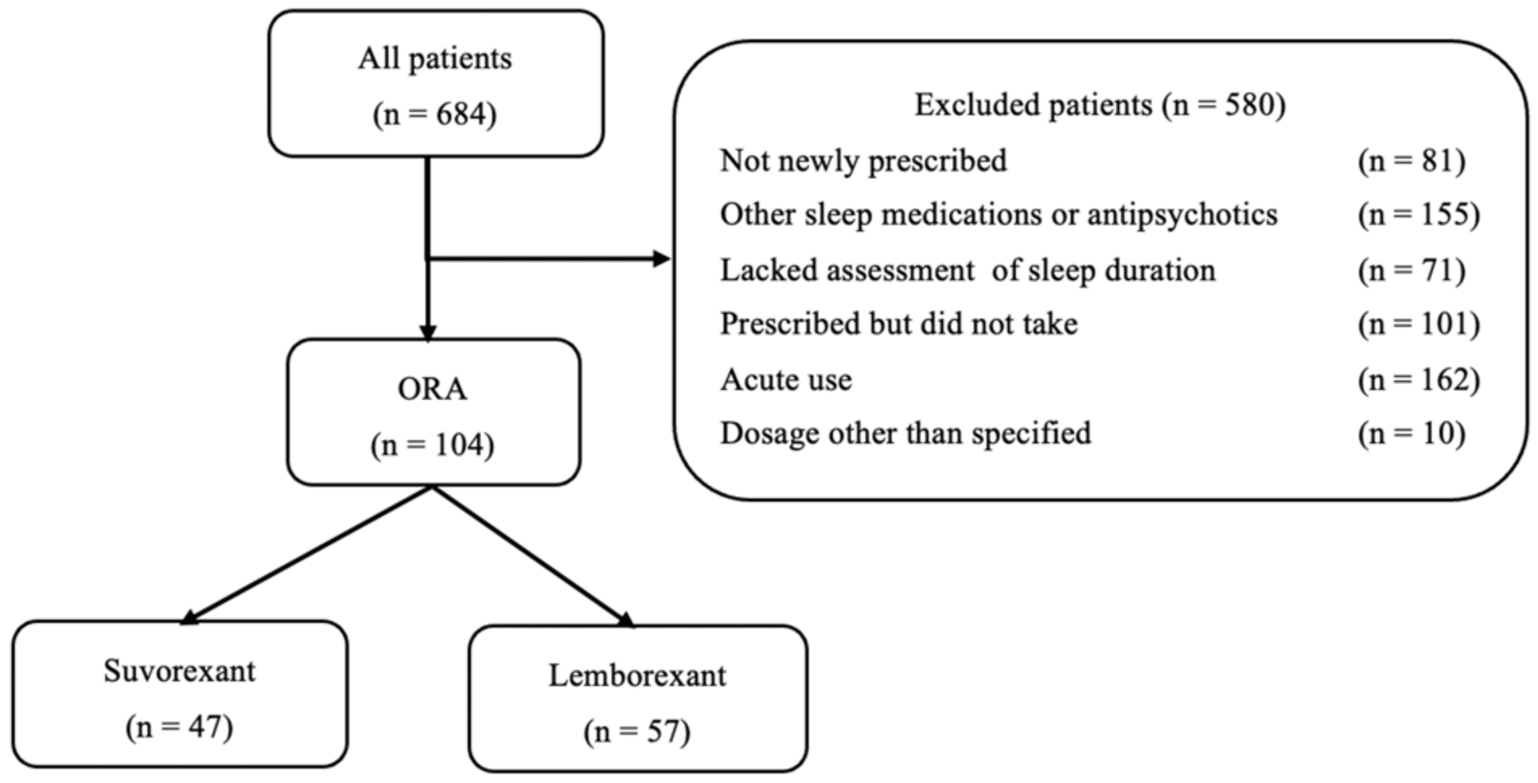
Flowchart of the patient selection process ORA: orexin receptor antagonists. This figure was prepared by the first author.

The type of study was a retrospective study. This study included patients who were newly prescribed suvorexant (15 mg) or lemborexant (5 mg) and hospitalized between April 1, 2022 and December 31, 2022 (n = 684). The drug regimen was chosen by the attending physician. Patients who were taking other sleep medications or antipsychotics (n = 155), those who did not undergo a sleep duration assessment on the day before the start of medication or at least one of the three days (n = 71), and those who used the medication during the acute phase were excluded from participation (n = 162). “Used the medication during the acute phase” was defined as patients who underwent surgical procedures, were admitted to the intensive care unit, or visited the emergency ward during the evaluation period. In addition, patients who were not newly prescribed the medication (n = 81), those who were not taking the medication (n = 101), and those who were not taking the medication before sleep (n = 10) were excluded. The study patients were 47 in the suvorexant group and 57 in the lemborexant group (Figure 1).

Outcomes

The primary endpoint was sleep duration. Sleep time was defined as the duration of sleep recorded in the electronic medical record, which included the day prior to starting the medication up to three days after starting the medication. Within-group comparisons for sleep duration were performed before and during the three days after taking the medication, and between-group comparisons were performed for each day after taking the medication. Sleep time was assessed by nurses who made rounds approximately every hour. Lights-out at the hospital was at 21:00, and sleep time was defined as the period following this. The secondary endpoint was the incidence of falls.

Data collection

Data collection included investigating patient background information such as age, sex, height, weight, days of hospitalization, days from admission to start of medication, diseases causing hospitalization, activities of daily living performance before hospitalization, coexisting disease, premedication (sleep and antipsychotic medications), and sleep time before medication use. Sleep duration during the first three days after starting medication was also evaluated. The relevant information was collected retrospectively using electronic medical records.

To evaluate sleep duration, nurses monitored patients during rounds and medication administration and recorded their sleep time in the electronic medical record. If the sleep duration was not recorded, the data were considered missing. The study period for falls was during hospital admission with continued oral medication.

Ethics approval

All procedures performed in studies involving human participants were in accordance with the ethical standards of the institutional and/or national research committee and with the 1964 Helsinki Declaration and its later amendments or comparable ethical standards. The study was approved by the Institutional Review Board of Ogaki Municipal Hospital (No. 20230427-10).

Data analysis

For the sleep duration endpoint, between-group comparisons were performed using Student's t-tests, whereas before/after comparisons were performed using paired t-tests with Bonferroni correction. For patient background variables such as sex, department of admission, and medications used, chi-square and Fisher's exact probability tests were employed, whereas differences in age, height, weight, length of hospitalization, and time between admission and start of medication were analyzed using Mann-Whitney U tests. Statistical significance was set at P < 0.05.

## Results

Table 1 summarizes the patient characteristics, which did not differ significantly between the two examined groups. There were no significant differences in premedications between the two groups, and eszopiclone was the most common premedication in both. Antipsychotic premedication rates were similar between the two groups. Patients in the suvorexant group had a slightly longer sleep duration three days before administration than patients in the lemborexant group, which persisted after treatment. Pre-medication consisted of zolpidem in two cases, eszopiclone in seven cases, and risperidone in one case in the suvorexant group. In the lemborexant group, 10 cases received zolpidem and eszopiclone, and ramelteon, suvorexant, brotizolam, quetiapine, and risperidone were administered to one case each.

**Table 1:** Characteristics of patients in the suvorexant and lemborexant groups ADL: activities of daily living, SD: standard deviation. n=number of patients and (%) percentage of patients, n=median of value and [range]=interquartile range of value, or n=average ± standard deviation. a: Mann–Whitney U test, b: chi-square test, c: Fisher’s exact test, and d: Student's t-test. Statistical significance was set at P < 0.05.

Characteristic	Suvorexant group		Lemborexant group		P-value
	(n=47)		(n=57)		
	n/(%), median {range} or average ± SD		n/(%), median {range} or average ± SD		
Age (years)	81	{74-88}	79	{72-88}	0.728^a^
Sex					0.292^b^
Male	26	(54.2)	25	(43.9)	
Female	22	(45.8)	32	(56.1)	
Height (cm)	155.4	{150.0-162.0}	153.5	{148.0-165.0}	0.636^ a^
Weight (kg)	51.7	{41.7-58.9}	48.9	{41.3-62.5}	0.827^a^
Sequence of events					
Days of hospitalization	24	{14-33}	24	{15-38}	0.421^a^
Days from admission to start of medication	6	{3-11}	8	{3-18}	0.652^a^
Diseases causing hospitalization					0.305^b^
Infection	11	(23.4)	13	(22.8)	
Cancer	5	(10.6)	9	(15.8)	
Trauma	1	(2.1)	6	(10.5)	
Heart disease	8	(17.0)	5	(8.8)	
Cerebral infarction	8	(17.0)	5	(8.8)	
Other	15	(31.9)	19	(33.3)	
ADL before hospitalization					0.755^b^
Independence	35	(74.5)	39	(68.4)	
Partial support	8	(17.0)	13	(22.8)	
Full support	4	(8.5)	5	(8.8)	
Coexisting disease					
Dementia	10	(21.3)	8	(14.0)	0.357^b^
Schizophrenia	6	(12.8)	2	(3.5)	0.084^b^
Depression	1	(2.1)	6	(10.5)	0.084^b^
Neurosis	3	(6.4)	6	(10.5)	0.436^b^
Premedication					
Sleep medications					0.395^c^
Benzodiazepine	0	(0.0)	1	(1.8)	
Zolpidem	2	(4.2)	1	(1.8)	
Eszopiclone	7	(14.6)	10	(17.5)	
Ramelteon	0	(0.0)	1	(1.8)	
Suvorexant	-		3	(5.3)	
Lemborexant	0	(0.0)	-		
Antipsychotic medications					1.000^c^
Quetiapine	0	(0.0)	1	(1.8)	
Risperidone	1	(2.1)	1	(1.8)	
Sleep time before dose (hour)					
3 days	5.16	±1.86	4.75	±1.95	0.348^d^
2 days	4.63	±2.14	4.88	±2.04	0.578^d^
1 day	4.52	±2.15	4.55	±1.98	0.937^d^

**Figure 2:**
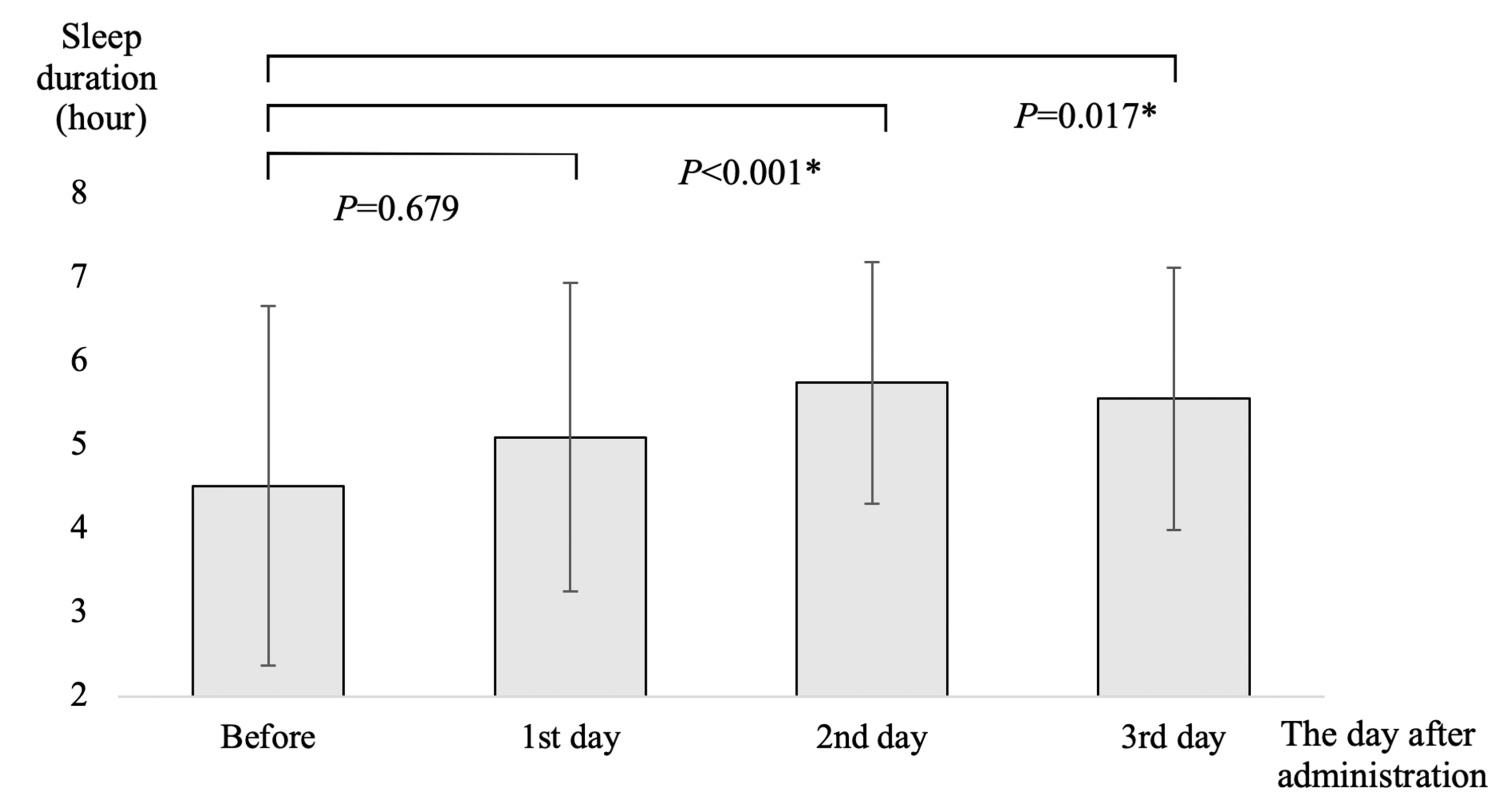
Sleep duration following suvorexant administration. X-axis: The day after administration. Y-axis: Sleep duration (in hours). Data are presented as the average sleep duration, with the error bars indicating the standard deviation. Statistical analysis was performed using a paired t-test with Bonferroni correction. *: P<0.05.

**Figure 3:**
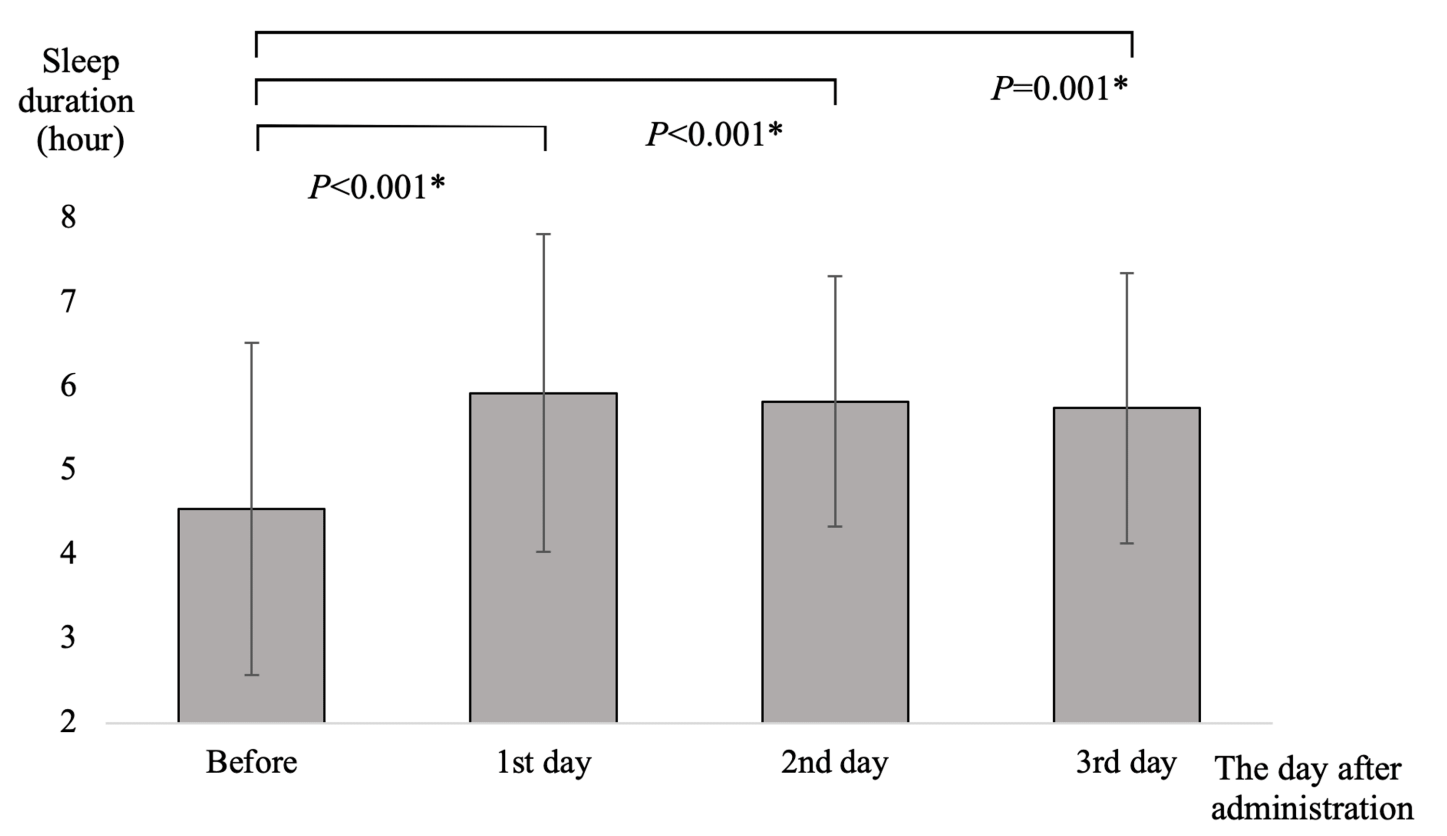
Sleep duration following lemborexant administration. X-axis: The day after administration. Y-axis: Sleep duration (in hours). Data are presented as the average sleep duration, with the error bars indicating the standard deviation. Statistical analysis was performed using a paired t-test with Bonferroni correction. *: P<0.05.

In the suvorexant group, sleep duration was prolonged on days 2 and 3 compared with that in the pre-dose period (Figure 2). Patients in the lemborexant group exhibited a significantly longer sleep duration than the pre-dose group on all three days after the first day of treatment (Figure 3). On the first day of treatment, the lemborexant group had a significantly longer sleep duration than the suvorexant group (suvorexant group 5.10 ± 1.81 h, lemborexant group 5.97 ± 1.90 h; P = 0.026). Sleep duration did not differ between the two groups after the second and third days of treatment and exhibited similar trends (Table 2). Falls were not observed in the suvorexant group (0/47: 0.0%), whereas three (3/57 5.3%) falls occurred in the lemborexant group on days 5, 11, and 30. Although the fall rate was higher in the lemborexant group versus the suvorexant group, this difference was not significant (P=0.248).

**Table 2:** Transition of the sleep duration before and after suvorexant and lemborexant administration SD: standard deviation. Statistical analysis was performed by Student's t-test. Statistical significance was set at P < 0.05.

	Suvorexant group	Lemborexant group	P-value
	Average ± SD	Average ± SD	
Sleep time (hour)			
1 day before dose	4.52 ±2.15	4.55 ±1.97	0.937
1 day after dose	5.10 ±1.84	5.93 ±1.90	0.026
2 days after dose	5.75 ±1.44	5.83 ±1.50	0.772
3 days after dose	5.66 ±1.57	5.75 ±1.59	0.539
Difference before to after (hours)			
The first day and last day	0.58 ±2.77	1.38 ±2.46	0.122

## Discussion

We compared the effects of suvorexant and lemborexant on sleep duration and the fall rate and found that both medications could effectively improve sleep duration; treatment with lemborexant markedly prolonged sleep duration on the first day, compared with suvorexant. The incidence of falls was higher in the lemborexant group versus the suvorexant group; however, the difference was not significant.

Lemborexant is more effective than suvorexant in terms of sleep onset latency. And lemborexant is equally effective in improving sleep maintenance and total sleep time [18] and is recommended in many clinical situations in Japan [19]. In addition, reports indicate that switching from suvorexant to lemborexant improves difficulties with falling asleep [20] and prolongs sleep duration [21], which is resemble to our findings. This is because lemborexant exerts a stronger inhibitory effect against OX2R than suvorexant; OX2R is a key receptor for sleep regulation [14]. Additionally, lemborexant has a shorter Tmax, resulting in a shorter time to sleep onset, and longer T1/2 [16,17] which ensures delayed systemic elimination. Pharmacokinetically, lemborexant exerts a greater effect than suvorexant does. Our findings suggest that lemborexant is more effective than suvorexant on the first day of use; additionally, the immediate efficacy of lemborexant is expected to enhance patient satisfaction and reduce the risk of switching to high-risk drugs, such as BZDs.

Despite the increased falls in the lemborexant group versus the suvorexant group, this difference was not significant. Suvorexant and lemborexant are not muscle relaxants [22,23] and they minimally impact the rate of falls when compared with other sleep medications [9,24]. However, as mentioned earlier, lemborexant can exert a relatively robust inhibitory effect on sleep-related receptors [14,25,26] and has a longer T1/2; these factors could have resulted in reduced daytime alertness. The differences in the incidence of falls between lemborexants and suvorexants require further research.

The onset of insomnia can be triggered by various factors [27], including hospitalization [28] or environmental changes [29]. In this study, organizational factors (i.e., symptomatic mental disorders such as dementia and depression) were investigated, with no significant differences found between the two groups but there was a bias toward schizophrenia and depression, which may have influenced this study. However, the sleep duration before administration of the drug was similar, so background effects were unlikely to be significant. All pre-medications were administered at least three days prior to starting the study; considering their half-life, they did not influence the results.

This study has some limitations. First, sleep duration may not have been accurately measured as the primary endpoint was the sleep time recorded by nurses in an electronic medical record. And there may have been a case of middle-of-the-night awakening. Ideally, sleep duration should be measured while detecting brain waves and body movements. Additionally, this was a subjective evaluation based on nurses’ judgment, and there were no uniform evaluation standards. This was also a retrospective study. The environment, hospitalization status, and other factors may have differed substantially among patients taking drugs, and it was difficult to obtain retrospective information. However, the sleep conditions of the patients were similar, and it is believed that this did not markedly impact our results. Additionally, fall rates are occasionally underestimated; inpatients, under the constant supervision of nurses, have limited freedom of activity, resulting in a low fall rate [30]. Consequently, the findings of this study may not be generalizable to the outpatient population. Nevertheless, we believe that the results show that there are considerable aspects to consider when comparing these two drugs.

## Conclusions

Based on the results of this study, lemborexant appears to be more effective than suvorexant in prolonging sleep duration from the first day of drug administration. Although lemborexant may increase the risk of falls, both lemborexant and suvorexant are typically considered to exert a limited effect on fall risk, compared with other sleep medications. These findings indicate that among ORAs, lemborexant showed quicker improvement in sleep duration, while suvorexant may be beneficial for patients concerned with fall risk.
